# Characterization of a Novel Bispecific Antibody That Activates T Cells *In Vitro* and Slows Tumor Growth *In Vivo*

**DOI:** 10.1089/mab.2019.0035

**Published:** 2019-12-06

**Authors:** Olesya Chornoguz, Catherine N. Leettola, Karen Leander, Kerry Brosnan, Eva Emmell, Mark L. Chiu, Sandra Santulli-Marotto

**Affiliations:** Janssen Biotherapeutics, Janssen R&D, Spring House, Pennsylvania.

**Keywords:** CD3 redirection, bispecific antibodies, mechanism of action

## Abstract

Although CD3 T cell redirecting antibodies have been successfully utilized for the treatment of hematological malignancies (blinatumomab), the T cell signaling pathways induced by these molecules are incompletely understood. To gain insight into the mechanism of action for T cell redirection antibodies, we created a novel murine CD3xEpCAM bispecific antibody that incorporates a silent Fc to dissect function and signaling of murine CD8 OT1 T cells upon stimulation. T cell-mediated cytotoxicity, cytokine secretion, expression of activation markers, and proliferation were directly induced in T cells treated with the novel CD3xEpCAM bispecific molecule *in vitro* in the presence of epithelial cell adhesion molecule (EpCAM) expressing tumor cells. Nanostring analysis showed that CD3xEpCAM induced a gene expression profile that resembled antigen-mediated activation, although the magnitude was lower than that of the antigen-induced response. In addition, this CD3xEpCAM bispecific antibody exhibited *in vivo* efficacy. This is the first study that investigates both *in vitro* and *in vivo* murine CD8 T cell function and signaling induced by a CD3xEpCAM antibody having a silent Fc to delineate differences between antigen-independent and antigen-specific T cell activation. These findings expand the understanding of T cell function and signaling induced by CD3 redirection bispecific antibodies and may help to develop more efficacious CD3 redirection therapeutics for cancer treatment, particularly for solid tumors.

## Introduction

CD3 redirection bispecific antibodies represent a cancer immunotherapy strategy that redirects T cells to kill tumor cells regardless of antigen specificity through direct engagement of CD3ɛ.^(1–4)^ An example of successful clinical application of a bispecific T cell redirection antibody is the CD3xCD19 bispecific T cell engager (BiTE), blinatumomab,^(5,6)^ which was approved by the Food and Drug Administration for treatment of acute lymphoblastic leukemia in 2014. Although another T cell redirection antibody CD3xEpCAM, catumaxomab, was previously approved for clinical use in Europe in 2009, it has since been withdrawn from the market.^(7,8)^ There are currently 45^(9)^ CD3-based T cell redirection bispecific antibodies including BCMAxCD3, Her2xCD3, CEAxCD3, and PSMAxCD3^(10–12)^ being tested in clinical trials for treatment of solid and hematological tumors. Because T cell redirection antibodies are amenable to large-scale manufacturing,^(10,11,13)^ and orchestrate tumor cell killing in an MHC/TCR (major histocompatibility complex/T cell antigen receptor)-independent manner,^(1)^ which increases the number of T cells able to respond, they are a promising modality for cancer immunotherapy.

Studies demonstrate that CD3 redirection bispecific antibodies bring T cells and target cells together, which leads to CD3 clustering, indicative of immune synapse formation, culminating in T cell activation and effector function.^(4,5,10)^ The immune synapse triggered by the CD3 redirection bispecific antibodies resembles that induced by cognate antigen in the MHC/TCR interaction^(1)^ in that clustering of CD3, Lck, and perforin takes place as well as ZAP70 translocation and CD45 exclusion.^(14)^ Subsequently, T cell proliferation is induced and is accompanied by acquisition of cytotoxic effector function via the perforin/granzyme B pathway supporting the hypothesis that the therapeutic effect of CD3 redirection bispecific antibodies is due to T cell activation, as has been demonstrated *in vitro* using T cell lines and primary T cells.^(1,5,14–16)^ Although suggestive, the majority of these studies focused on synapse comparison for MHC/TCR-activated versus CD3-redirection-bispecific-antibody-activated T cell lines^(1,14)^ rather than on bispecific antibody activation in primary T cells.^(12,16,17)^ Only a few studies address T cell function mediated by CD3 redirection antibody compared with MHC/TCR-activated primary CD8 T cells.^(12,16,17)^ To address this, a novel CD3xEpCAM T cell redirection bispecific antibody composed of an anti-mouse CD3ɛ paired with an anti-human epithelial cell adhesion molecule (EpCAM) binding arm and a functionally silent Fc has been constructed to characterize murine CD8 T cell activation and function in the absence of Fc effector function. The functional Fc on CD3 redirection molecules could cause CD3 clustering and T cell activation in the absence of target tumor cells,^(9)^ which could lead to an unwanted toxicity. Therefore, we created a molecule with a silent Fc. Furthermore, this antibody on the murine IgG2aσ backbone with anti-murine CD3 enables *in vivo* studies in syngeneic tumor models, which will help to improve the understanding of CD3 redirection antibodies mechanism of action in physiological settings. Currently, there is only one other CD3 redirection molecule with a silent Fc^(17)^ amenable for use in syngeneic tumor models with wild-type mice.

This bispecific molecule has enabled a comprehensive characterization of CD3 redirection bispecific antibody activation of CD8 T cells to understand how it differs from antigen-mediated activation of using the ovalbumin (OVA)-specific OT1 T cells.^(18,19)^ We report that *in vitro* CD3xEpCAM-mediated CD8 T cell activation, as measured by cytotoxicity, cytokine secretion, proliferation, and expression of T cell activation markers, had a similar profile and kinetics to cognate antigen-mediated activation. However, CD3xEpCAM-mediated T cell activation was lower in magnitude compared with antigen-mediated activation. The CD3xEpCAM bispecific antibody exhibited *in vivo* activity as it significantly reduced growth of human EpCAM expressing B16F10 tumors. This *in vivo* efficacy is exclusively due to CD3 and EpCAM engagement since the Fc has been engineered to be functionally silent.^(20)^ Furthermore, although this molecule elicited relatively low levels of T cell activation *in vitro*, this did not appear to be predictive of the characteristics required for *in vivo* efficacy in a murine melanoma model.

## Materials and Methods

### Antibody construction and production

The amino acid sequences of the 4–7 V_H_ and V_L_ of the antibody were reverse translated, and eukaryotic signal peptide sequences were appended at the N-termini. The resulting DNA fragments were synthesized (Integrated DNA Technologies, Coralville, IA) and cloned into modified pcDNA 3.1 vectors containing the mouse IgG2a sigma T370K/K409R heavy chain constant region (vector digested with *Hin*dIII and *Dra*III) or the mouse kappa light chain constant region (vector digested with *Hin*dIII and *Eco*RI) using In-Fusion cloning (Takara Bio USA, Mountain View, CA). Anti-mouse CD3ɛ was produced by cloning the V_H_ and V_L_ sequences of clone 2C11^(21)^ into vectors containing the mouse IgG2a sigma F405L heavy chain and mouse lambda light chain, respectively. The null arm control, anti-respiratory syncytial virus (RSV),^(22)^ was produced by cloning the V_H_ and V_L_ sequences of clone B21M into vectors containing the mouse IgG2a sigma F405L heavy chain and mouse kappa light chain, respectively. The anti-RSV V_H_ segment was also cloned into a vector containing the mouse IgG2a sigma T370K/K409R heavy chain.^(20)^ Final constructs were verified by sequencing (Genewiz, South Plainfield, NJ).

Proteins were expressed by transfecting Expi293F cells with heavy and light chain plasmid at a ratio of 1:3, respectively, using an ExpiFectamine Kit according to the manufacturer's recommendations (Thermo Fisher Scientific, Carlsbad, CA). Protein was expressed for ∼5 days at 37°C in a humidified carbon dioxide incubator with shaking. Cells were harvested by centrifugation at 2000 × *g* and supernatant was filtered through 0.2 μm filter.

Antibody protein was purified using a 5 mL HiTrap MabSelect SuRe column (GE Life Sciences, Uppsala, Sweden) equilibrated in phosphate-buffered saline (PBS) and run at 4 mL/min. Protein was eluted using 50 mM citrate, pH 3.5. Eluted protein was immediately applied to a HiPrep 26/10 desalting column equilibrated in PBS and run at 8 mL/min. Purified protein was pooled. To remove aggregate, purified protein was loaded onto an SRT-10C-S300 21.2 × 300 mm column (Sepax Technologies, Inc.) equilibrated in PBS and run at 5 mL/min. Fractions containing purified monomeric protein were pooled and concentrated using Amicon Ultra Centrifugal filter units (EMD Millipore, Billerica, MA).

### Bispecific antibody production

Bispecific antibodies were produced from purified parental proteins using controlled Fab-arm exchange (cFAE) according to the established methods.^(23,24)^ In a mouse framework, the F405L mutation on the heavy chain of one parental antibody and the T370K, K409R mutations on the heavy chain of another parental antibody permit efficient cFAE.^(25)^ CD3xEpCAM and CD3xnull were produced by mixing purified 2C11 mIgG2a sigma F405L with a 5% excess of purified anti-EpCAM or B21M mIgG2a sigma T370K/K409R; a 5% excess was used limit the presence of any residual bivalent anti-CD3ɛ. EpCAMxnull was produced by mixing B21M mIgG2a sigma F405L with an equivalent amount of anti-EpCAM mIgG2a sigma T370K/K409R. 2-Mercaptoethylamine dissolved in 1 M Tris, pH 7.5, was added to the mixed proteins to a final concentration of 75 mM, and proteins were incubated at 31°C for 5 hours. Proteins were then dialyzed against 1 × PBS, pH 7.2. CD3xEpCAM and CD3xnull were polished by loading onto an SRT-10C-S300 21.2 × 300 mm column (Sepax Technologies, Inc.) equilibrated in PBS and run at 5 mL/min to remove aggregate. Fractions containing monomeric protein were pooled. Bispecific antibody was quantitated by hydrophobic interaction chromatography high performance liquid chromatography (HPLC) using a TSKgel Butyl-NPR column (Tosoh Bioscience, Griesheim, Germany) equilibrated in 1.5 M AmSO_4_, 0.1 M NaHPO_4_, pH 6.5. Thirty micrograms of protein was injected onto the column and run at 0.5 mL/min with a gradient to 0.1 M NaHPO_4_, pH 6.5, over 25 minutes. All proteins contained >94% bispecific antibody. All proteins contained minimal aggregate, as assessed by size exclusion chromatography-HPLC by injecting 100 μg protein onto a TSKgel G3SW_XL_ column (Tosoh Bioscience) run at 1 mL/min in PBS. Proteins were >95% pure as assessed by nonreducing and reducing sodium dodecyl sulfate–polyacrylamide gel electrophoresis. Endotoxin was <1 EU/mg as measured using Pyrotell-T reagent on a Pyros Kinetix Flex instrument according to the manufacturer's protocol (Associates of Cape Cod, Inc.).

### Nanostring gene expression analysis

Purified OT1 T cells were plated at 1 × 10^6^ per well into a 12-well plate and activated with either mature dendritic cell (DC) loaded with SIINFEKL peptide or CD3xEpCAM or CD3xnull in the presence of B16F10/EpCAM target cell for 24, 48, and 72 hours. Antibodies were added to cultures at 10 μg/mL. DC to T cell and B16F10/EpCAM to T cell ratio was 1:10. T cells were harvested at every time point and cell pellets were frozen in RLT buffer with 2-ME for subsequent RNA isolation. RNA was isolated using RNeasy Plus Mini RNA isolation kit from Qiagen and quantified using DropSense Trinean spectrophotometer. Fifty nanograms of RNA per sample was used for Nanostring gene expression analysis that was conducted according to the manufacturer's protocol. The custom gene expression panel consisted of 30 T cell activation/effector function genes and 4 housekeeping genes (Supplementary Table S1). Nanostring data were analyzed using nSolver 3.0 Software.

### Chemicals and antibodies

All reagents and chemicals were purchased from Sigma–Aldrich, unless noted otherwise. All flow antibodies for mouse T cell analysis were purchased from BioLegend. The following mouse antibodies were used: CD69 PerCP-Cy5.5, CD8 FITC, and CD44 BV510. Anti-mouse immunoglobulin G (IgG) allophycocyanin (APC)-conjugated secondary antibody was purchased from Jackson ImmunoResearch. Violet proliferation dye V450 was purchased from BD, and near-infrared (IR) live–dead dye was purchased from Invitrogen.

### Flow cytometry

For all flow cytometry, experiments cells were stained with near-IR live–dead dye (Invitrogen) at room temperature for 5 minutes in PBS and then incubated with a mouse Fc-Block (BioLegend) bovine serum albumin-containing BD flow buffer (BD Pharmingen) at 4°C. Subsequently, all samples were stained with either the antibody cocktail or the isotype control for 30 minutes at 4°C and then washed and fixed in BD Cytofix (BD Pharmingen). Samples were run on the BD LSRFortessa (BD Biosciences) and then analyzed using the FloJo Software. The gate was set on live cells, doublets were excluded, and isotype control antibody stained cells were used to set gates for negative and positive populations.

### Cell lines

B16F10 and E.G7-OVA cell lines were obtained from Janssen cell repository in Spring House, PA. E.G7-OVA were cultured in Roswell Park Memorial Institute (RPMI) with 10% fetal bovine serum (FBS) in the presence of 0.4 mg/mL of G418. B16F10 were cultured in Dulbecco's modified Eagle medium with 10% FBS. B16F10/EpCAM cells stably expressing human EpCAM were generated by transfecting B16F10 with a pCMV vector encoding human EpCAM (Sino Biological) with a hygromycin resistance gene. B16F10 cells were transfected with EpCAM pCMV plasmid using Lipofectamine 3000 (Invitrogen) and then were put on selection media containing 1.8 mg/mL hygromycin to select for B16F10 cells that were stably transfected with human EpCAM. B16F10/EpCAM stable cell line was maintained in 1.8 mg/mL hygromycin.

### Mice

OT1 and WT C57BL/6 mice were obtained from Jackson Laboratory and maintained at Janssen's Spring House animal facility. All animal protocols were approved by Janssen Institutional Animal Care and Use Committee.

### T cell activation assays

OT1 T cells were purified from spleens and lymph nodes of OT1 mice using a negative selection CD8 T cell purification kit from Miltenyi. T cell purity was 85%–95% in all experiments as verified by CD3/CD8 surface expression using flow cytometry. C57BL/6 DCs used for T cell activation assays were purchased from Astarte. To mature DC for T cell activation assays, a previously described DC maturation protocol was used.^(26,27)^ DCs were cultured overnight in 125 ng/mL lipopolysaccharide, 50 ng/mL interferon gamma (IFNγ), 50 ng/mL interleukin (IL)-4, and 50 ng/mL granulocyte-macrophage colony-stimulating factor (PeproTech). Mature DCs were then loaded with 10 ng/mL of SIINFEKL peptide in serum-free RPMI for 3 hours at 37°C. For T cell activation assays, purified OT1 were combined with mature DCs that were either SIINFEKL loaded or unloaded at 10:1 ratio. Alternatively, OT1 T cells were combined with either 10 μg/mL of CD3xEpCAM or CD3xnull antibody and B16F10/EpCAM target cells at 10:1 E:T ratio. OT1 T cells activated by either antibodies or DC were cultured for 24, 48, and 72 hours, and T cell activation was assayed using flow cytometry.

### Cytotoxic T lymphocyte assays

DELFIA (Perkin-Elmer) Cell Cytotoxicity assay was conducted according to the manufacturer's protocol. Briefly, OT1 T cells were purified from spleens and lymph nodes of OT1 mice and seeded into six-well plates at 1.5 × 10^6^ cells/mL. Mature DCs loaded with SIINFEKL peptide were added to OT1 T cells at 1:10 ratio and were cultured for 48–72 hours. Activated OT1 T cells were counted and plated into a round bottom 96-well plate. Then, BATDA-labeled E.G7-OVA, B16F10, or B16F10/EpCAM cells were added to T cells in a 96-well plate. B16F10/EpCAM-labeled target cells were added with either 10 μg/mL of CD3xEpCAM or CD3xnull antibodies. T cells were incubated with target cells in a total volume of 200 μL for 4 hours. After 4-hour incubation, 20 μL of supernatants was harvested and combined with 200 μL of europium solution, and fluorescence was read on EnVision 96-well plate reader.

### Multiplex cytokine analysis

Multiplex cytokine analysis was conducted using Meso Scale Discovery (MSD) 10-plex mouse proinflammatory panel according to the manufacturer's protocol. The 10-plex panel included the following cytokines/chemokines: IFNγ, IL-1β, IL-2, IL-4, IL-6, KC/GRO, IL-10, IL-12p70, and tumor necrosis factor alpha (TNF-α).

### Statistical analysis

For all flow data, the gates were set using either isotype stained or unstained controls. Quantitative analysis for flow data was performed on date exported from the FlowJo. Statistical analysis for flow, MSD, and *in vivo* tumor study was conducted in GraphPad Prism version 6. Statistical significance and p-values for MSD cytokine data and flow activation marker expression was performed using two-way analysis of variance (Holm–Sidak).

### B16F10/EpCAM *in vivo* tumor studies

All procedures were carried out in the vivarium at Janssen Research and Development, LLC (Spring House, PA). The facility is accredited by the Association for Assessment and Accreditation of Laboratory Animal Care.

A 5- to 7-week-old female C57Bl/6 mice were purchased from Horizon and were acclimated for 10–14 days before experimentation. Mice were assigned by weight to either treatment (CD3xEpCAM) or control (CD3xnull) groups. Eight mice per group were used, with the group body weight after assignment averaging 18 g. On day 0, 100 μL of B16F10/EpCAM cells (7.5 × 10^5^ cells/mouse) was implanted subcutaneously in the right flank of each mouse using a 25-gauge needle. Biweekly intraperitoneal antibody administration was started on day 1 and concluded after the fourth dose on day 10. Mice in CD3xEpCAM and CD3xNull groups received 60 μg per dose of corresponding monoclonal antibodies. Mice were observed daily for clinical signs. Biweekly body weights and tumor volumes were recorded using a three-dimensional subcutaneous tumor scanning device (TumorImager™; Biopticon, Princeton, NJ). At the termination of the study, mice were sacrificed by carbon dioxide asphyxiation.

## Results

### CD3xEpCAM antibody binds to murine CD3 and human EpCAM to redirect OT1 T cells

**FIG. 1.**
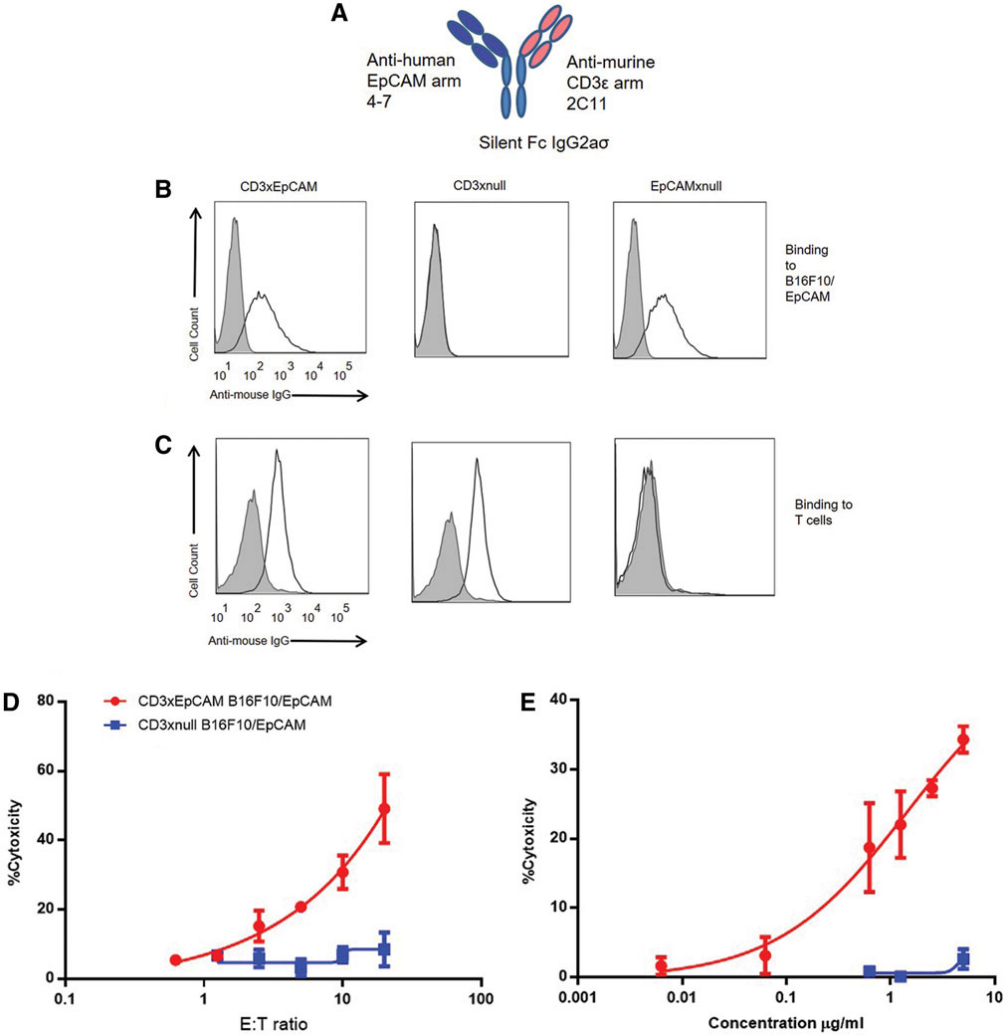
CD3xEpCAM murine bispecific antibody binds mouse CD3 and human EpCAM and redirected OT1 T cells to kill human EpCAM expressing B16F10. **(A)** CD3xEpCAM mouse bispecific antibody had anti-murine CD3ɛ arm (clone 2C11) and anti-human EpCAM arm (clones 4–7).^(15)^ **(B)** B16F10/EpCAM cells were incubated with 10 μg/mL of either CD3xEpCAM or CD3xnull and EpCAMxnull controls. Antibody binding to human EpCAM on B16F10 was detected using anti-mouse IgG APC secondary antibody. Secondary antibody stained cells only (gray-filled histograms) were used as a negative control, and open histograms denotes staining with CD3xEpCAM bispecific antibody of either B16F10/EpCAM **(B)** or OT1 T cells **(C)**. **(D, E)** The CD3xEpCAM antibody redirected OT1 T cells isolated from spleen and lymph nodes of OT1 mice and pre-activated with SIINFEKL peptide loaded C57BL/6 mature DC for 48–72 hours as demonstrated by BADTA/Eu-based CTL. **(D)** E:T ratio was varied in the presence of 10 μg/mL of either CD3xEpCAM (red circles) or CD3xnull negative control (blue squares). **(E)** The concentration of either CD3xEpCAM (red circles) or CD3xnull (blue circles) was varied from 0.006 to 5 μg/mL, and the E:T is kept constant at 10:1. The data are representative of three individual experiments. APC, allophycocyanin; CTL, cytotoxic T lymphocytes; DC, dendritic cell; EpCAM, epithelial cell adhesion molecule; IgG, immunoglobulin G.

To explore the mechanism of action for a CD3 redirection bispecific antibody in a murine model system, a CD3xEpCAM bispecific molecule was designed such that any functional activity would be mediated exclusively by engagement of the variable regions. This molecule was engineered to harbor a mouse IgG2aσ silent Fc (Fig. 1A), with one variable region arm specific for mouse CD3ɛ and the other arm specific for human EpCAM to permit targeting of murine tumor cells expressing human EpCAM. Murine IgG was chosen as the Fc for the architecture of the bispecific antibody because this bispecific antibody format offered the advantage of stability both *in vitro* and *in vivo* as well as enabling better production yield. Silent IgG2aσ Fc^(20)^ was chosen to allow interrogation of CD8 T cell function induced by the T cell redirection antibody without the confounding participation of Fc-mediated effector mechanisms.

The B16F10 cell line has been well documented as a poorly immunogenic melanoma cell line that grows aggressively *in vivo*.^(28,29)^ This melanoma cell line B16F10 was engineered to stably express human EpCAM, and the resulting B16F10/EpCAM cells were used to verify binding of the CD3xEpCAM to human EpCAM. To confirm that the CD3xEpCAM antibody binds to both mouse CD3 and human EpCAM, the CD3xEpCAM antibody was incubated with either mouse primary T cells or B16/EpCAM cells and then stained with anti-mouse IgG APC-conjugated secondary antibody. As shown in Figure 1B, only molecules harboring EpCAM specificity exhibited binding to the B16F10/EpCAM cells as no staining could be detected using the CD3xnull control. The ability of CD3xEpCAM to specifically bind to murine CD3 was confirmed using OT1 T cells (Fig. 1C). To verify that CD3xEpCAM can redirect antigen-specific T cells, OVA-specific T cells from OT1 mice were employed. The OT1 T cells respond specifically to targets displaying the OVA-derived SIINFEKL peptide^(18,19)^; however, in the presence of CD3xEpCAM, pre-activated OT1 T cells were able to kill the B16/EpCAM cells (Fig. 1D, E). Only the CD3xEpCAM, but not the CD3xnull antibody, redirected the OVA-specific OT1 T cells to kill OVA^-^ B16F10/EpCAM target cells indicating that the killing was T cell mediated, independent of TCR antigen specificity, and correlated with the dose of CD3xEpCAM (Fig. 1D, E). Therefore, CD3xEpCAM antibody binds to both mouse CD3ɛ and human EpCAM and redirects OT1 T cells to kill B16/EpCAM tumor cells in a target-specific manner by essentially changing the CD8 T cell specificity from OVA to EpCAM.

### CD3xEpCAM induces expression of T cell activation markers, cytokine secretion, and proliferation in OT1 T cells in the presence of EpCAM expressing tumor cells

**FIG. 2.**
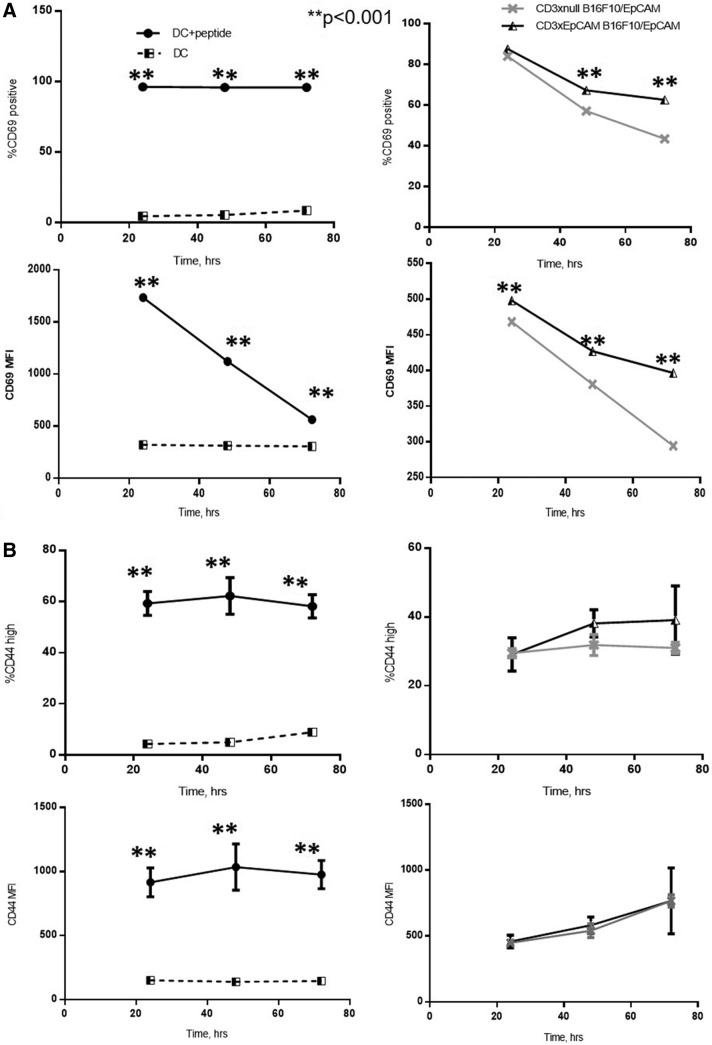
T cell activation induced by CD3xEpCAM is lower in magnitude compared with antigen-induced activation. OT1 T cells were purified from spleens and lymph nodes of OT1 mice and activated for 24, 48, or 72 hours, with either mature DC loaded with SIINFEKL or 10 μg/mL of CD3xEpCAM or CD3xnull with B16/EpCAM target cells at 10:1 ratio. The cells were harvested and stained with live–dead dye as well as for CD8, CD69, and CD44 at every time point. **(A)** Percent CD69-positive CD8-positive T cells and CD69 MFI for CD8-positive T cells activated as indicated above. **(B)** Percent CD44 high CD8-positive T cells and CD44 MFI for CD8-positive T cells activated as indicated above. The data are representative of three individual experiments. MFI, mean channel fluorescence.

To examine T cell activation potential and kinetics mediated by the CD3xEpCAM antibody, freshly isolated OT1 T cells were cocultured with B16/EpCAM tumor cells and analyzed for expression of CD69 and CD44 activation markers^(30–32)^ at 24, 48, and 72 hours (Fig. 2A, B). In parallel, SIINFEKL-loaded DCs were cocultured with OT1 T cells to provide a point of reference for expression kinetics of CD69 and CD44 under optimal conditions. As expected, OT1 T cells activated with SIINFEKL-loaded DC had a large population of CD69^+^ and CD44^high^ T cells. Activation of OT1 T cells by either CD3xEpCAM or CD3xnull in the presence of B16F10/EpCAM target cells led to an increase in the percentage of CD69^+^ and CD44^high^ cells (Fig. 2A); however, CD3xEpCAM-activated OT1 T cells had a significantly higher frequency of CD69^+^ cells accompanied by corresponding higher cell surface levels compared with CD3xnull-activated T cells. This was not the case for the CD44 expressing population as there were no statistically significant differences between frequency and cell surface expression levels in the CD44^high^ OT1 T cells that were activated with CD3xEpCAM or CD3xnull antibodies even in the presence of B16/EpCAM.

**FIG. 3.**
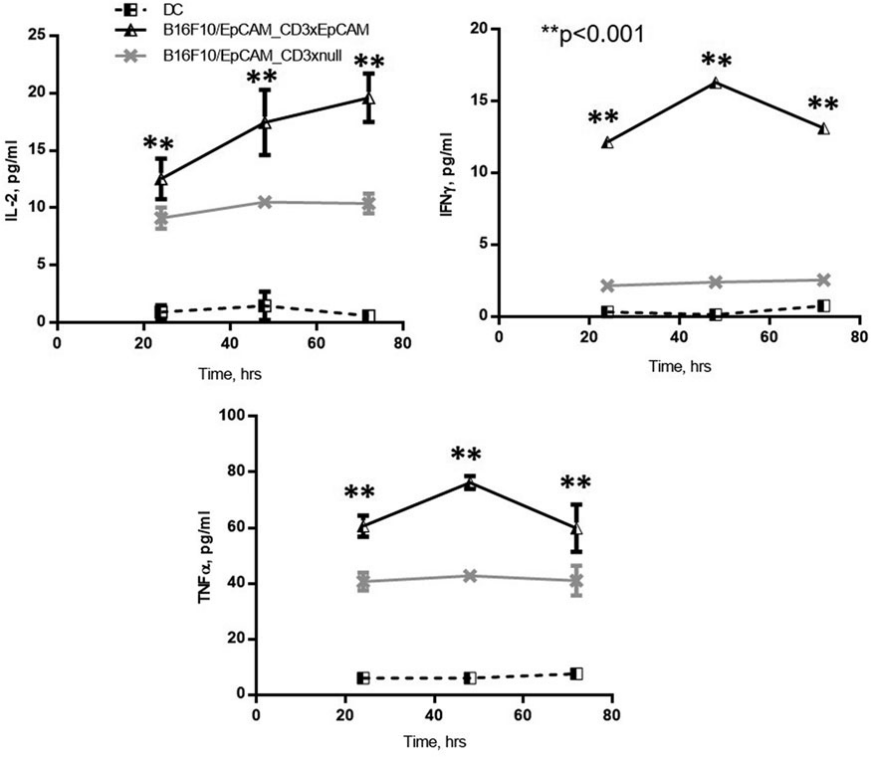
Cytokine secretion in OT1 T cells in response to CD3xEpCAM stimulation. Supernatants from the same sample as shown in Figure 2A were harvested and assayed for IL-2, IFNγ, and TNF-α using 10-plex Meso Scale Discovery. The data are representative of three individual experiments. IFNγ, interferon gamma; IL-2, interleukin 2; TNF-α, tumor necrosis factor alpha.

**FIG. 4.**
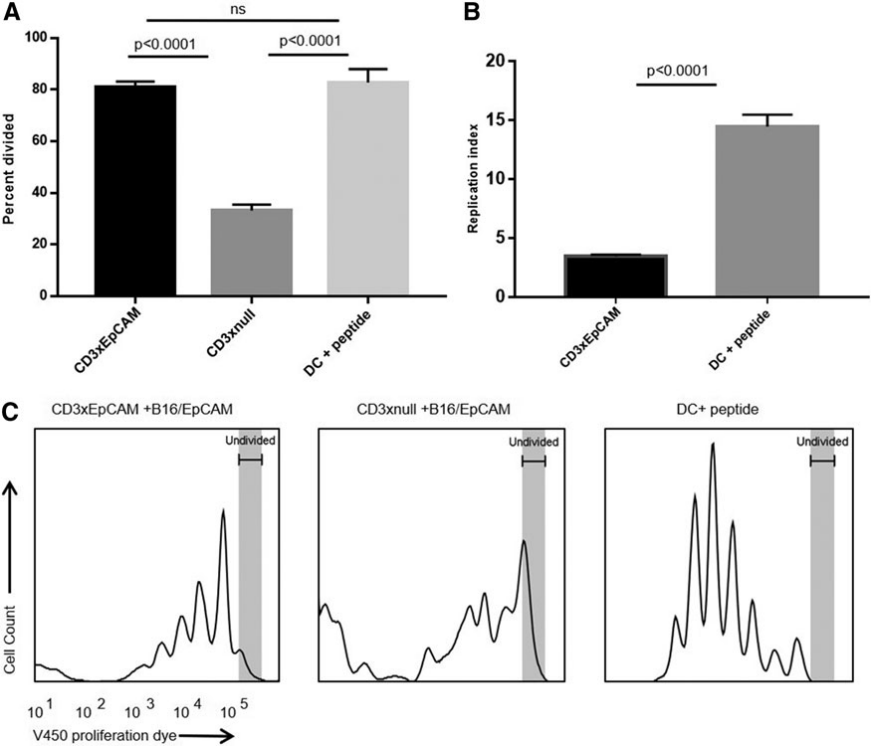
CD3xEpCAM antibody is induced OT1 T cell proliferation. To measure OT1 T cell proliferation, T cells were labeled with violet proliferation dye and activated with the same stimuli as shown in Figure 3 for 72 hours. To measure percentage, divided gates were set on viable T cells. **(A)** Percentage of T cells that divided in response to different stimuli plotted as a bar graph. **(B)** Replication index for CD3xEpCAM and DC plus SIINFEKL-stimulated T cells. **(C)** Flow cytometry gating strategy and representative flow plots for samples shown in **(A)** and **(B)**. The data are representative of three individual experiments. ns, non-significant.

To understand the relationship between the T cell phenotype and cytokine secretion, supernatants were harvested from T cells activated as shown in Figure 2 and analyzed for cytokine levels at 24, 48, and 72 hours. Both IFNγ and TNF-α cytokine secretion induced by CD3xEpCAM peaked at 48 hours (Fig. 3) possibly due to the peak of T cell activation being between 24 and 48 hours as shown by the kinetics of T cell activation markers in Figure 2. Levels of IL-2, IFNγ, and TNF-α were significantly higher at every time point in CD3xEpCAM-treated cultures compared with cytokine levels secreted in response to CD3xnull control (Fig. 3). As expected, levels of IL-2, IFNγ, and TNF-α were much lower when compared with the control SIINFEKL-loaded DC-activated T cells (Supplementary Fig. S1). Similarly, a higher frequency of OT1 T cells proliferated when activated by CD3xEpCAM compared with CD3xnull treatment of OT1 T cells cultured with B16/EpCAM (Fig. 4A). The frequency of OT1 T cells that had divided following the stimulation with either SIINFEKL-loaded mature DC or CD3xEpCAM was similar, although stimulation with DC plus SIINFEKL resulted in a higher replication index (Fig. 4B), which is defined as fold expansion over the culture time. This is not unexpected in that cognate antigen stimulation induces more potent proliferation compared with CD3xEpCAM. Stimulation with either CD3xEpCAM or CD3xnull resulted in upregulation of CD69 and CD44 T cell activation markers on naive OT1 T cells in the presence of B16/EpCAM cells; however, only the CD3xEpCAM bispecific could induce cytokine secretion and proliferation. These studies demonstrate that the CD3xEpCAM bispecific mediate T cell activation and effector function when in the presence of ligand-expressing tumor cells.

### The gene expression profile exhibited by OT1 T cells redirected by CD3xEpCAM resembles that observed during stimulation with cognate antigen

**FIG. 5.**
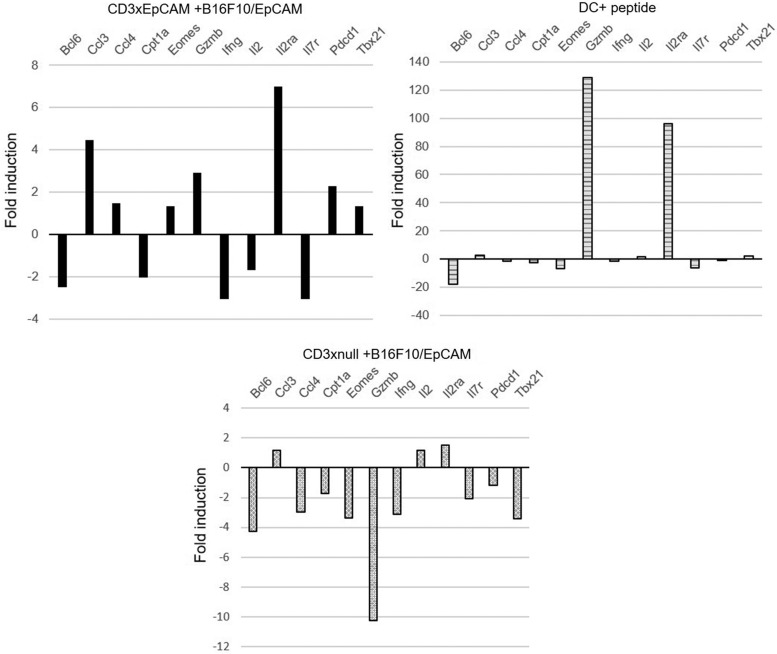
CD3xEpCAM-activated T cells expressed lower levels of T cell effector function genes. RNA was isolated and Nanostring gene expression analysis was conducted on OT1 T cells activated for 48 hours as shown in Figures 2 and 3, and Supplementary Figure S2.

To elucidate the activation signaling pathways induced by CD3xEpCAM, a custom Nanostring gene expression panel was created that consisted of genes expressed during T cell activation/effector function (Supplementary Table S1).^(33,34)^ This gene panel included transcription factors that drive T cell effector or memory differentiation such as *Tbx21*, *Eomes*, and *Bcl6*, as well as genes that are crucial for T cell proliferation and cytotoxicity such as *IL2ra*, *Il2*, *Ifng*, and *Gzmb*. This analysis of OT1 T cells was conducted using cells activated, by coculture with B16/EpCAM in the presence of CD3xEpCAM, CD3xnull bispecifics, or DC loaded with SIINFEKL as previously described. From these cultures, the nonadherent cells were harvested, which consisted almost entirely of T cells, as the tumor cells and DCs are adherent, then RNA was prepared from cultures at 24-, 48-, and 72-hour time points (Fig. 5; Supplementary Fig. S2). The fold change in gene expression was derived using RNA from unstimulated T cells that were incubated with media as the baseline for the indicated time points. This analysis revealed that at 48 hours CD3xEpCAM-activated OT1 T cells exhibited a gene expression profile that resembled that of OVA antigen-activated OT1 T cells, with the main difference being that the magnitude of the response for some of the genes such as IL-2 and GrzB was reproducibly 50- to 100-fold lower in CD3xEpCAM-treated T cells cocultured with B16/EpCAM (Fig. 5; Supplementary Fig. S2).

Notably, *GrzmB*, *Il2ra*, and *Ccl3* were reproducibly upregulated more than twofold over unstimulated T cells in CD3xEpCAM-treated cocultures of B16/EpCAM and OT1 T cells; *GrzB* and *Il2ra* genes are crucial for the effector response in antigen-activated T cells (Fig. 5; Supplementary Fig. S2). Some genes were upregulated to a similar degree (*ccl3* and *tbx21*) in response to both antigen- and CD3xEpCAM-induced T cell activation. In addition, *Eomes* was uniquely upregulated 1.34-fold in the CD3xEpCAM-treated T cells. Since the activity of T-bet is in part regulated posttranslationally,^(35)^ it is difficult to interpret the meaning of changes in the T-bet transcript levels. Treatment with the CD3xnull antibody did not elicit any significant increase in expression of T cell effector genes at any of the time points studied (Fig. 5; Supplementary Fig. S2).

### The CD3xEpCAM bispecific antibody reduces tumor growth and extends survival of B16F10/EpCAM tumor-bearing animals

**FIG. 6.**
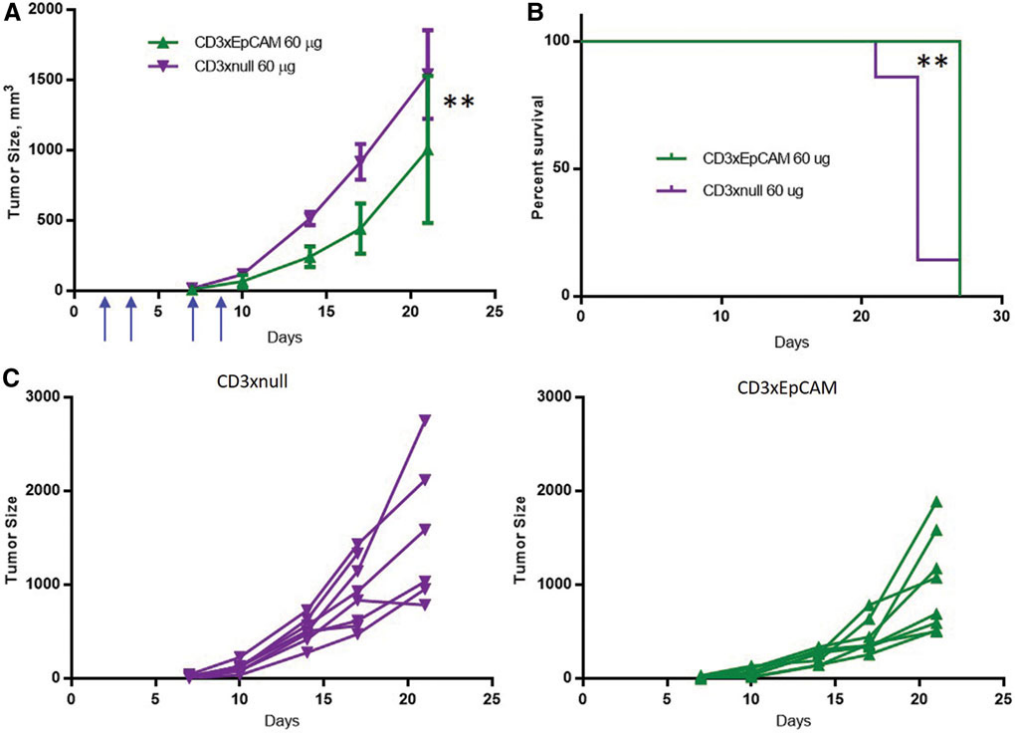
CD3xEpCAM treatment increased survival and reduced tumor growth of B16F10/EpCAM-treated mice. Sixteen C57BL/6 mice were implanted with B16F10/EpCAM tumors. Eight mice were treated biweekly with 60 μg of CD3xEpCAM antibody per dose (green line) or 60 μg of CD3xnull antibody over the course of 2 weeks. Blue arrows indicate antibody injections. **(A)** Average tumor growth of CD3xEpCAM- and CD3xnull-treated mice. Error bars are standard error of the mean. **(B)** Survival plot of CD3xEpCAM- and CD3xnull-treated groups. **(C)** Spider plots for CD3xnull (purple lines) or CD3xEpCAM (green lines)-treated mice. **p < 0.004 by the Wilcoxon sign-rank test for tumor size growth and the Mantel–Cox test for survival. The data are representative of two individual experiments.

The CD3xEpCAM bispecific antibody was demonstrated to activate CD8 T cells *in vitro*; however, the question remained as to whether this would translate into an ability to interfere with tumor growth *in vivo*. To determine whether treatment with CD3xEpCAM was sufficient to impact tumor growth *in vivo*, C57BL/6 mice were implanted with B16F10/EpCAM tumor cells and then treated with either CD3xEpCAM or CD3xnull control bispecific antibody as described. As shown in Figure 6, treatment with the CD3xEpCAM bispecific antibody significantly reduced the growth of tumors (Fig. 6A, C) and extended survival of B16F10/EpCAM tumor-bearing mice (Fig. 6B) compared with CD3xnull-treated animals. This indicates that both specificities of the bispecific antibody are required for reduction of tumor growth and, based on the *in vitro* CD8 T cell data, it is highly likely that redirected CD8 T cells play a role. These data indicate that the CD3xEpCAM bispecific antibody can redirect T cells to kill human EpCAM expressing target tumor cells both *in vitro* and *in vivo*.

## Discussion

This study is the first to comprehensively characterize the activation properties of a novel CD3xEpCAM bispecific antibody with a silent Fc and its impact on antigen-specific murine CD8 T cell function. While a previous study used 2C11 anti-murine CD3 and 4–7 anti-human EpCAM for creation of a BiTE,^(15)^ to our knowledge, this is the first fully murine CD3xEpCAM antibody on a mouse IgG2aσ silent Fc architecture. Even though BiTEs have demonstrated efficacy in clinic, their *in vivo* half-life is about 1 hour.^(9,15)^ CD3 redirection molecules with a silent Fc have a significantly longer half-life of several weeks *in vivo*,^(17)^ which makes them better suited for clinical use. This CD3xEpCAM bispecific molecule with a silent Fc has successfully redirected pre-activated OT1 T cells to kill B16F10 cells that express human EpCAM. Additionally, this T cell redirection antibody induced cytokine secretion and proliferation upon treatment of freshly isolated OT1 T cells. In accordance with the *in vitro* activity, the CD3xEpCAM bispecific antibody significantly reduced tumor growth in mice bearing B16F10/EpCAM tumors.

Although unable to match the potency of an antigen-presenting cell, the CD3xEpCAM bispecific induced enough signal to affect the activation state of freshly isolated OT1 T cells. This was shown by increased cytokine production and proliferative response as well as cell surface marker expression. There are many differences in these two methods of T cell stimulation with the most notable being the cooperative action of co-stimulatory and adhesion molecules expressed on APCs that enhance their activation capacity.

In the case of T cell redirection antibodies, it is known that the magnitude of T cell activation induced can be modulated by engineering binding affinity, structural modifications such as spacing between binding domains, or changing antigen binding site to enable recognition of an alternate epitope.^(13,36)^ Binding affinities for each arm of the CD3xEpCAM have been previously published; the K_d_ value for 2C11 anti-murine CD3 has been reported to be 90 nM and the K_d_ for 4–7 anti-human EpCAM has been reported to be 50 nM.^(15)^ These affinities are much tighter than those generally seen for TCR-peptide-MHC interactions and for OVA the K_d_ reported for OT1 TCR bound K^b^ + OVA peptide is 5.9 μM.^(37)^ Although not the sole factor, difference in levels of T cell activation induced by CD3xEpCAM antibody compared with OVA-loaded DCs was not due to weaker affinity of antibody binding to CD3ɛ and EpCAM compared with MHC/TCR interaction. Because stimulation with APCs is so different from stimulation with the CD3xEpCAM, a reasonable comparison cannot be made; however, the efficiency and consistency of the APC–T cell interaction lends itself to a reliable reference point for T cell activation. It is conceivable that by optimizing binding affinity and kinetics of binding to CD3 and to EpCAM, the *in vitro* and *in vivo* activity of the CD3xEpCAM bispecific may be enhanced. That the CD3xEpCAM bispecific molecule could activate CD8^+^ T cells *in vitro* regardless of TCR specificity and reduce tumor growth *in vivo* even though optimal antibody architecture and affinity was not identified is in itself remarkable.

Perhaps only a fraction of OT1 T cells responded to CD3xEpCAM-mediated stimulation as reflected by the low frequency of CD3xEpCAM-activated T cells that exhibited upregulation of CD69 and CD44 (Fig. 2) compared with SIINFEKL-activated OT1. This is further supported by the low frequency of OT1 T cells that proliferated in response to the CD3xEpCAM bispecific antibody in coculture with B16/EpCAM cells. This phenomenon could be representative of immune response in young adult or pediatric cancer patients who have a higher fraction of naive CD8 T cells^(38,39)^ with higher threshold for activation. In senior adult cancer patients, it is likely that more of their CD8 T cells are antigen experienced^(40)^ and may be more readily activated by T cell redirection antibodies.

The *in vivo* data demonstrated that CD3xEpCAM significantly reduced tumor growth and prolonged survival of B16/EpCAM tumor-bearing mice (Fig. 6). While exploring combination therapies to enhance *in vivo* efficacy of CD3xEpCAM is beyond the scope of the work presented here, it should be noted that *in vivo* potency could potentially be further enhanced by combining CD3xEpCAM with either anti-PD1 or other checkpoint inhibitors. A recent study^(41)^ demonstrated that anti-PD-L1 enhances the potency of a CD3xCD33 antibody *in vitro*, thus combining CD3xEpCAM with anti-PD1 could enhance CD3xEpCAM efficacy *in vivo* and would be an informative experiment.

Our finding that the CD3xEpCAM bispecific demonstrates *in vivo* efficacy concomitant with relatively lower *in vitro* activity suggests that rigorous T cell activation is not necessary to achieve *in vivo* efficacy. Furthermore, this finding was recently replicated in a study that used a fully murine CD3 redirection antibody that had relatively low level of *in vitro* cytotoxicity, yet exhibited significant *in vivo* efficacy.^(17)^ In fact, strong activation of CD8 T cells may cause adverse events, such as cytokine release syndrome (CRS). Therefore, a T cell redirection antibody that induces T cell effector function that is just rigorous enough to achieve *in vivo* efficacy, in the absence of CRS may provide the optimal therapeutic window for efficacious cancer treatment. This optimal therapeutic window may be achieved by the low affinity anti-CD3 arm^(9)^ and modulating the tumor antigen affinity to tailor it to the antigen expression levels.

Although our *in vitro* studies were designed to specifically address the response of CD8 T cells to CD3 redirection antibody engagement, it is highly likely that *in vivo*, CD4 T cells also play a role in tumor eradication. This is supported by several studies^(17,42–44)^ demonstrating that CD4 T cells play a prominent role in the anti-tumor immune response. Thus, the CD4 T cell contribution could explain the enhanced *in vivo* activity compared with *in vitro* activation and function using purified CD8 T cells.

In conclusion, this is the first study that used CD3xEpCAM on mouse IgG2aσ framework with a silent Fc to specifically characterize CD8 T cell activation and effector function in response to T cell redirection antibody stimulation. The CD3xEpCAM bispecific antibody was shown to be capable of directly stimulating CD8 T cells *in vitro* and reducing tumor growth in B16/EpCAM tumor-bearing mice. Engagement of the OT1 T cells by CD3xEpCAM appears to engage a subset of the same cellular signaling pathways that operate during conventional MHC/TCR-mediated T cell activation. The characterization of this tool CD3 redirection bispecific molecule sets the stage for additional studies to delve into the *in vivo* mechanism of action of this bispecific to better understand how it is impacting the T cells to limit tumor growth. Altogether, the data from this study shed light on mechanism of action for CD3 redirection bispecific antibodies and can be used for design and testing of novel efficacious T cell redirection molecules.

## Supplementary Material

Supplemental data

Supplemental data

Supplemental data

## References

[1] Offner S, Hofmeister R, Romaniuk A, Kufer P, and Baeuerle PA: Induction of regular cytolytic T cell synapses by bispecific single-chain antibody constructs on MHC class I-negative tumor cells. Mol Immunol 2006;43:763–771 16360021 10.1016/j.molimm.2005.03.007

[2] Perez P, Hoffman RW, Shaw S, Bluestone JA, and Segal DM: Specific targeting of cytotoxic T cells by anti-T3 linked to anti-target cell antibody. Nature 1985;316:354–356 3160953 10.1038/316354a0

[3] Staerz UD, Kanagawa O, and Bevan MJ: Hybrid antibodies can target sites for attack by T cells. Nature 1985;314:628–631 2859527 10.1038/314628a0

[4] Wong JT, and Colvin RB: Bi-specific monoclonal antibodies: Selective binding and complement fixation to cells that express two different surface antigens. J Immunol 1987;139:1369–1374 3112233

[5] Wong R, Pepper C, Brennan P, Nagorsen D, Man S, and Fegan C: Blinatumomab induces autologous T-cell killing of chronic lymphocytic leukemia cells. Haematologica 2013;98:1930–1938 23812940 10.3324/haematol.2012.082248 PMC3857012

[6] Zugmaier G, Gökbuget N, Klinger M, Viardot A, Stelljes M, Neumann S, Horst HA, Marks R, Faul C, Diedrich H, Reichle A, Brüggemann M, Holland C, Schmidt M, Einsele H, Bargou RC, and Topp MS: Long-term survival and T-cell kinetics in relapsed/refractory ALL patients who achieved MRD response after blinatumomab treatment. Blood 2015;126:2578–2584 26480933 10.1182/blood-2015-06-649111 PMC4671107

[7] Shen J, and Zhu Z: Catumaxomab, a rat/murine hybrid trifunctional bispecific monoclonal antibody for the treatment of cancer. Curr Opin Mol Ther 2008;10:273–284 18535935

[8] European Medicines Agency: Removab withdrawal of the marketing authorisation in the European Union. 2017 Available at: https://www.ema.europa.eu/en/documents/public-statement/public-statement-removab-withdrawal-marketing-authorisation-european-union_en.pdf (accessed 1121, 2019)

[9] Labrijn AF, Janmaat ML, Reichert JM, and Parren P: Bispecific antibodies: A mechanistic review of the pipeline. Nat Rev Drug Discov 2019;18:585–608 31175342 10.1038/s41573-019-0028-1

[10] Jachimowicz RD, Borchmann S, and Rothe A: Multi-specific antibodies for cancer immunotherapy. BioDrugs 2014;28:331–343 24638872 10.1007/s40259-014-0091-4

[11] Zhang X, Yang Y, Fan D, and Xiong D: The development of bispecific antibodies and their applications in tumor immune escape. Exp Hematol Oncol 2017;6:12 28469973 10.1186/s40164-017-0072-7 PMC5414286

[12] Oberst MD, Fuhrmann S, Mulgrew K, Amann M, Cheng L, Lutterbuese P, Richman L, Coats S, Baeuerle PA, and Hammond SA: CEA/CD3 bispecific antibody MEDI-565/AMG 211 activation of T cells and subsequent killing of human tumors is independent of mutations commonly found in colorectal adenocarcinomas. mAbs 2014;6:1571–1584 25484061 10.4161/19420862.2014.975660 PMC4622052

[13] Wu Z, and Cheung NV: T cell engaging bispecific antibody (T-BsAb): From technology to therapeutics. Pharmacol Ther 2018;182:161–175 28834699 10.1016/j.pharmthera.2017.08.005 PMC5785550

[14] Li J, Stagg NJ, Johnston J, Harris MJ, Menzies SA, DiCara D, Clark V, Hristopoulos M, Cook R, Slaga D, Nakamura R, McCarty L, Sukumaran S, Luis E, Ye Z, Wu TD, Sumiyoshi T, Danilenko D, Lee GY, Totpal K, Ellerman D, Hötzel I, James JR, and Junttila TT: Membrane-proximal epitope facilitates efficient T cell synapse formation by anti-FcRH5/CD3 and is a requirement for myeloma cell killing. Cancer Cell 2017;31:383–395 28262555 10.1016/j.ccell.2017.02.001 PMC5357723

[15] Schlereth B, Kleindienst P, Fichtner I, Lorenczewski G, Brischwein K, Lippold S, da Silva A, Locher M, Kischel R, Lutterbüse R, Kufer P, and Baeuerle PA: Potent inhibition of local and disseminated tumor growth in immunocompetent mouse models by a bispecific antibody construct specific for Murine CD3. Cancer Immunol Immunother 2006;55:785–796 16187083 10.1007/s00262-005-0082-x PMC11029878

[16] Ishiguro T, Sano Y, Komatsu SI, Kamata-Sakurai M, Kaneko A, Kinoshita Y, Shiraiwa H, Azuma Y, Tsunenari T, Kayukawa Y, Sonobe Y, Ono N, Sakata K, Fujii T, Miyazaki Y, Noguchi M, Endo M, Harada A, Frings W, Fujii E, Nanba E, Narita A, Sakamoto A, Wakabayashi T, Konishi H, Segawa H, Igawa T, Tsushima T, Mutoh H, Nishito Y, Takahashi M, Stewart L, ElGabry E, Kawabe Y, Ishigai M, Chiba S, Aoki M, Hattori K, and Nezu J: An anti-glypican 3/CD3 bispecific T cell-redirecting antibody for treatment of solid tumors. Sci Transl Med 2017;9:pii: 10.1126/scitranslmed.aal4291 28978751

[17] Benonisson H, Altıntaş I, Sluijter M, Verploegen S, Labrijn AF, Schuurhuis DH, Houtkamp MA, Verbeek JS, Schuurman J, and van Hall T: CD3-bispecific antibody therapy turns solid tumors into inflammatory sites but does not install protective memory. Mol Cancer Ther 2018;18:312–322 30381448 10.1158/1535-7163.MCT-18-0679

[18] Hogquist KA, Jameson SC, Heath WR, Howard JL, Bevan MJ, and Carbone FR: T cell receptor antagonist peptides induce positive selection. Cell 1994;76:17–27 8287475 10.1016/0092-8674(94)90169-4

[19] Salem ML, Kadima AN, Zhou Y, Nguyen CL, Rubinstein MP, Demcheva M, Vournakis JN, Cole DJ, and Gillanders WE: Paracrine release of IL-12 stimulates IFN-gamma production and dramatically enhances the antigen-specific T cell response after vaccination with a novel peptide-based cancer vaccine. J Immunol 2004;172:5159–5167 15100252 10.4049/jimmunol.172.9.5159

[20] Tam SH, McCarthy SG, Armstrong AA, Somani S, Wu SJ, Liu X, Gervais A, Ernst R, Saro D, Decker R, Luo J, Gilliland GL, Chiu ML, and Scallon BJ: Functional, biophysical, and structural characterization of human IgG1 and IgG4 Fc variants with ablated immune functionality. Antibodies 2017;6:12 10.3390/antib6030012 PMC6698826 31548527

[21] Leo O, Foo M, Sachs DH, Samelson LE, and Bluestone JA: Identification of a monoclonal antibody specific for a murine T3 polypeptide. Proc Natl Acad Sci USA 1987;84:1374–1378 2950524 10.1073/pnas.84.5.1374 PMC304432

[22] Canziani GA, Melero JA, and Lacy ER: Characterization of neutralizing affinity-matured human respiratory syncytial virus F binding antibodies in the sub-picomolar affinity range. J Mol Recogn 2012;25:136–146 10.1002/jmr.2149 22407977

[23] Labrijn AF, Meesters JI, de Goeij BE, van den Bremer ET, Neijssen J, van Kampen MD, Strumane K, Verploegen S, Kundu A, Gramer MJ, van Berkel PH, van de Winkel JG, Schuurman J, and Parren PW: Efficient generation of stable bispecific IgG1 by controlled Fab-arm exchange. Proc Natl Acad Sci USA 2013;110:5145–5150 23479652 10.1073/pnas.1220145110 PMC3612680

[24] Labrijn AF, Meesters JI, Priem P, de Jong RN, van den Bremer ET, van Kampen MD, Gerritsen AF, Schuurman J, and Parren PW: Controlled Fab-arm exchange for the generation of stable bispecific IgG1. Nat Protoc 2014;9:2450–2463 25255089 10.1038/nprot.2014.169

[25] Labrijn AF, Meesters JI, Bunce M, Armstrong AA, Somani S, Nesspor TC, Chiu ML, Altintaş I, Verploegen S, Schuurman J, and Parren PWHI: Efficient generation of bispecific murine antibodies for pre-clinical investigations in syngeneic rodent models. Sci Rep 2017;7:2476 28559564 10.1038/s41598-017-02823-9 PMC5449386

[26] Sittig SP, de Vries IJM, and Schreibelt G: Primary human blood dendritic cells for cancer immunotherapy-tailoring the immune response by dendritic cell maturation. Biomedicines 2015;3:282–303 28536413 10.3390/biomedicines3040282 PMC5344227

[27] Sun HH, Zhou DF, and Zhou JY: The role of DCs in the immunopathogenesis of chronic HBV infection and the methods of inducing DCs maturation. J Med Virol 2016;88:13–20 26104380 10.1002/jmv.24306

[28] Fujiwara S, Nagai H, Shimoura N, Oniki S, Yoshimoto T, and Nishigori C: Intratumoral CD4+ T lymphodepletion sensitizes poorly immunogenic melanomas to immunotherapy with an OX40 agonist. J Invest Dermatol 2014;134:1884–1892 24468748 10.1038/jid.2014.42

[29] Hart IR: The selection and characterization of an invasive variant of the B16 melanoma. Am J Pathol 1979;97:587–600 507192 PMC2042430

[30] Conde M, Montaño R, Moreno-Aurioles VR, Ramirez R, Sanchez-Mateos P, Sanchez-Madrid F, and Sobrino F: Anti-CD69 antibodies enhance phorbol-dependent glucose metabolism and Ca2+ levels in human thymocytes. Antagonist effect of cyclosporin A. J Leukoc Biol 1996;60:278–284 8773590 10.1002/jlb.60.2.278

[31] Dianzani U, Bragardo M, Tosti A, Ruggeri L, Volpi I, Casucci M, Bottarel F, Josè Feito M, Bonissoni S, and Velardi A: CD44 signaling through p56lck involves lateral association with CD4 in human CD4+ T cells. Int Immunol 1999;11:1085–1092 10383941 10.1093/intimm/11.7.1085

[32] Hebert E: Endogenous lectins as cell surface transducers. Biosci Rep 2000;20:213–237 11092246 10.1023/a:1026484722248

[33] Veldman-Jones MH, Brant R, Rooney C, Geh C, Emery H, Harbron CG, Wappett M, Sharpe A, Dymond M, Barrett JC, Harrington EA, and Marshall G: Evaluating robustness and sensitivity of the NanoString technologies nCounter platform to enable multiplexed gene expression analysis of clinical samples. Cancer Res 2015;75:2587–2593 26069246 10.1158/0008-5472.CAN-15-0262

[34] Winham SJ, Mehner C, Heinzen EP, Broderick BT, Stallings-Mann M, Nassar A, Vierkant RA, Hoskin TL, Frank RD, Wang C, Denison LA, Vachon CM, Frost MH, Hartmann LC, Aubrey Thompson E, Sherman ME, Visscher DW, Degnim AC, and Radisky DC: NanoString-based breast cancer risk prediction for women with sclerosing adenosis. Breast Cancer Res Treat 2017;166:641–650 28798985 10.1007/s10549-017-4441-z PMC5668350

[35] Chornoguz O, Hagan RS, Haile A, Arwood ML, Gamper CJ, Banerjee A, and Powell JD: mTORC1 promotes T-bet phosphorylation to regulate Th1 differentiation. J Immunol 2017;198:3939–3948 28424242 10.4049/jimmunol.1601078 PMC5458608

[36] Mandikian D, Takahashi N, Lo AA, Li J, Eastham-Anderson J, Slaga D, Ho J, Hristopoulos M, Clark R, Totpal K, Lin K, Joseph SB, Dennis MS, Prabhu S, Junttila TT, and Boswell CA: Relative target affinities of T-cell-dependent bispecific antibodies determine biodistribution in a solid tumor mouse model. Mol Cancer Ther 2018;17:776–785 29339550 10.1158/1535-7163.MCT-17-0657

[37] Stone JD, Chervin AS, and Kranz DM: T-cell receptor binding affinities and kinetics: Impact on T-cell activity and specificity. Immunology 2009;126:165–176 19125887 10.1111/j.1365-2567.2008.03015.x PMC2632691

[38] Czesnikiewicz-Guzik M, Lee WW, Cui D, Hiruma Y, Lamar DL, Yang ZZ, Ouslander JG, Weyand CM, and Goronzy JJ: T cell subset-specific susceptibility to aging. Clin Immunol 2008;127:107–118 18222733 10.1016/j.clim.2007.12.002 PMC2435295

[39] Wertheimer AM, Bennett MS, Park B, Uhrlaub JL, Martinez C, Pulko V, Currier NL, Nikolich-Žugich D, Kaye J, and Nikolich-Žugich J: Aging and cytomegalovirus infection differentially and jointly affect distinct circulating T cell subsets in humans. J Immunol 2014;192:2143–2155 24501199 10.4049/jimmunol.1301721 PMC3989163

[40] Goronzy JJ, Fang F, Cavanagh MM, Qi Q, Weyand CM: Naive T cell maintenance and function in human aging. J Immunol 2015;194:4073–4080 25888703 10.4049/jimmunol.1500046 PMC4452284

[41] Laszlo GS, Gudgeon CJ, Harrington KH, and Walter RB: T-cell ligands modulate the cytolytic activity of the CD33/CD3 BiTE antibody construct, AMG 330. Blood Cancer J 2015;5:e340 26295610 10.1038/bcj.2015.68 PMC4558592

[42] Ott PA, Hu Z, Keskin DB, Shukla SA, Sun J, Bozym DJ, Zhang W, Luoma A, Giobbie-Hurder A, Peter L, Chen C, Olive O, Carter TA, Li S, Lieb DJ, Eisenhaure T, Gjini E, Stevens J, Lane WJ, Javeri I, Nellaiappan K, Salazar AM, Daley H, Seaman M, Buchbinder EI, Yoon CH, Harden M, Lennon N, Gabriel S, Rodig SJ, Barouch DH, Aster JC, Getz G, Wucherpfennig K, Neuberg D, Ritz J, Lander ES, Fritsch EF, Hacohen N, and Wu CJ: An immunogenic personal neoantigen vaccine for patients with melanoma. Nature 2017;547:217–221 28678778 10.1038/nature22991 PMC5577644

[43] Takeuchi Y, Tanemura A, Tada Y, Katayama I, Kumanogoh A, Nishikawa H: Clinical response to PD-1 blockade correlates with a sub-fraction of peripheral central memory CD4+ T cells in patients with malignant melanoma. Int Immunol 2018;30:13–22 29294043 10.1093/intimm/dxx073

[44] Wang D, Aguilar B, Starr R, Alizadeh D, Brito A, Sarkissian A, Wang D, Aguilar B, Starr R, Alizadeh D, Brito A, Sarkissian A, Ostberg JR, Forman SJ, and Brown CE: Glioblastoma-targeted CD4+ CAR T cells mediate superior antitumor activity. JCI Insight 2018;3:pii: 10.1172/jci.insight.99048 PMC6012522 29769444

