# Supplementary material for: Characterization of a Novel Bispecific Antibody That Activates T Cells In Vitro and Slows Tumor Growth In Vivo
Source: Monoclon Antib Immunodiagn Immunother. 2019 Dec 6;38(6):242–54. doi: 10.1089/mab.2019.0035 (PMC6918852; doi:10.1089/mab.2019.0035)
Supplement: Supplemental data [file Suppl_TableS1.pdf]

Supplementary Table S1. Nanostring Gene panel

| *Gene name* | *Accession no.* | *Class name* |
|---|---|---|
| Bcl6 | NM_009744.3 | Endogenous |
| Card11 | NM_175362.2 | Endogenous |
| Ccl3 | NM_011337.1 | Endogenous |
| Ccl4 | NM_013652.1 | Endogenous |
| Cd8a | NM_001081110.2 | Endogenous |
| Cpt1a | NM_013495.1 | Endogenous |
| Cxcl15 | NM_011339.2 | Endogenous |
| Eomes | NM_010136.2 | Endogenous |
| Foxp3 | NM_054039.2 | Endogenous |
| Fyn | NM_008054.2 | Endogenous |
| Gata3 | NM_008091.3 | Endogenous |
| Gzmb | NM_013542.2 | Endogenous |
| Havcr2 | NM_134250.2 | Endogenous |
| Icosl | NM_015790.3 | Endogenous |
| Ifng | NM_008337.1 | Endogenous |
| Il10 | NM_010548.1 | Endogenous |
| Il12a | NM_008351.1 | Endogenous |
| Il12b | NM_001303244.1 | Endogenous |
| Il2 | NM_008366.2 | Endogenous |
| Il2ra | NM_008367.2 | Endogenous |
| Il7r | NM_008372.3 | Endogenous |
| Klrg1 | NM_016970.1 | Endogenous |
| Lck | NM_001162433.1 | Endogenous |
| Nfkbib | NM_010908.4 | Endogenous |
| Nfkbie | NM_008690.3 | Endogenous |
| Pdcd1 | NM_008798.1 | Endogenous |
| Pfkp | NM_019703.2 | Endogenous |
| Slc2a1 | NM_011400.3 | Endogenous |
| Slc5a1 | NM_019810.4 | Endogenous |
| Tbx21 | NM_019507.2 | Endogenous |
| Actb | NM_007393.1 | Housekeeping |
| Polr1b | NM_009086.2 | Housekeeping |
| Tbp | NM_013684.3 | Housekeeping |
| Tubb4a | NM_009451.3 | Housekeeping |

This table provides the list of all genes measured in every Nanostring experiment. Four genes were housekeeping genes used for normalization.
