# Supplementary material for: Characterization of a Novel Bispecific Antibody That Activates T Cells In Vitro and Slows Tumor Growth In Vivo
Source: Monoclon Antib Immunodiagn Immunother. 2019 Dec 6;38(6):242–54. doi: 10.1089/mab.2019.0035 (PMC6918852; doi:10.1089/mab.2019.0035)
Supplement: Supplemental data [file Suppl_FigS1.pdf]

Supplementary Data

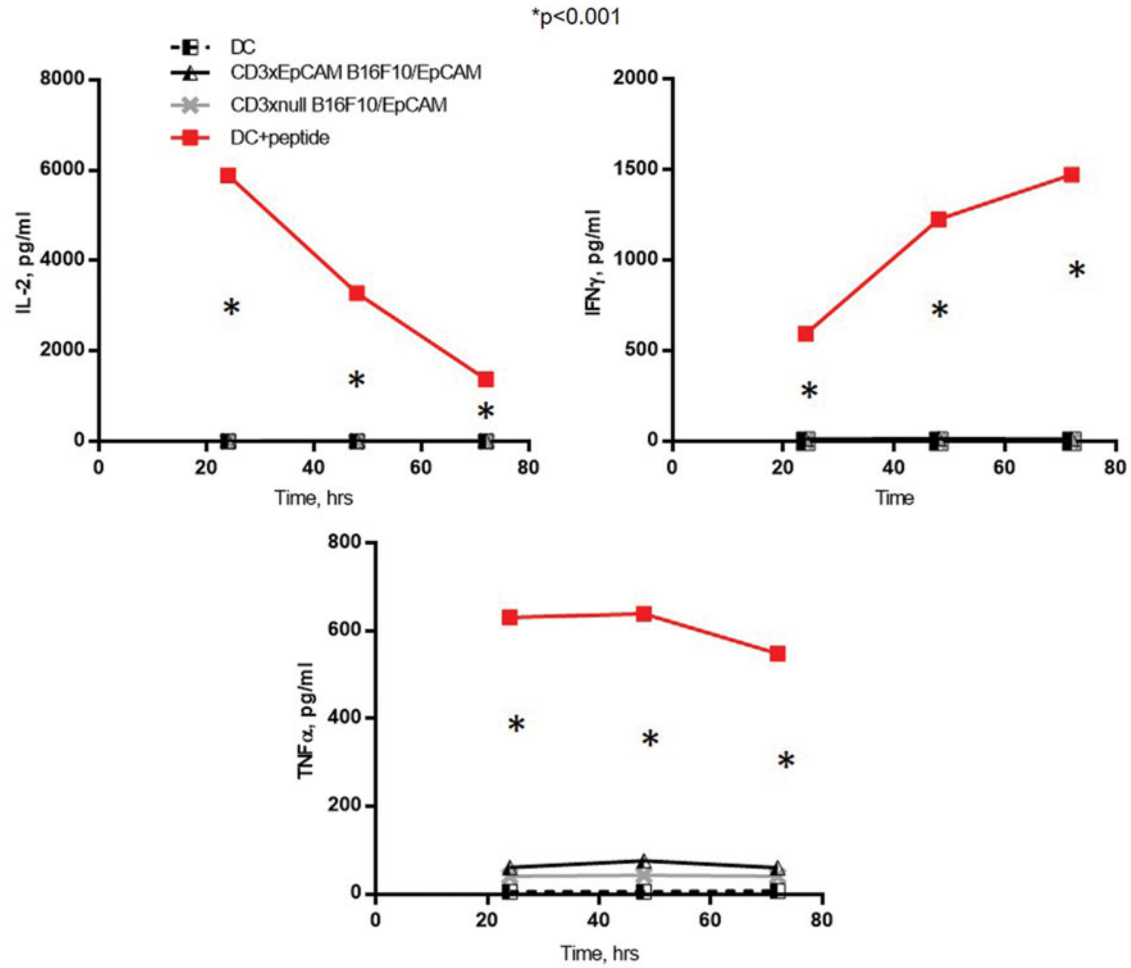

SUPPLEMENTARY FIG. S1. OT1 T cells were activated for 24, 48, and 72 hours as described in Figures 3 and 5. RNA was isolated at every time point and used for Nanostring gene expression analysis. Gene expression was compared with unstimulated OT1 T cells. *X*-axis lists different genes that were investigated and *y*-axis is the fold change in transcript levels over unstimulated T cells. The data are representative of two individual experiments.
