# Supplementary material for: Characterization of a Novel Bispecific Antibody That Activates T Cells In Vitro and Slows Tumor Growth In Vivo
Source: Monoclon Antib Immunodiagn Immunother. 2019 Dec 6;38(6):242–54. doi: 10.1089/mab.2019.0035 (PMC6918852; doi:10.1089/mab.2019.0035)
Supplement: Supplemental data [file Suppl_FigS2.pdf]

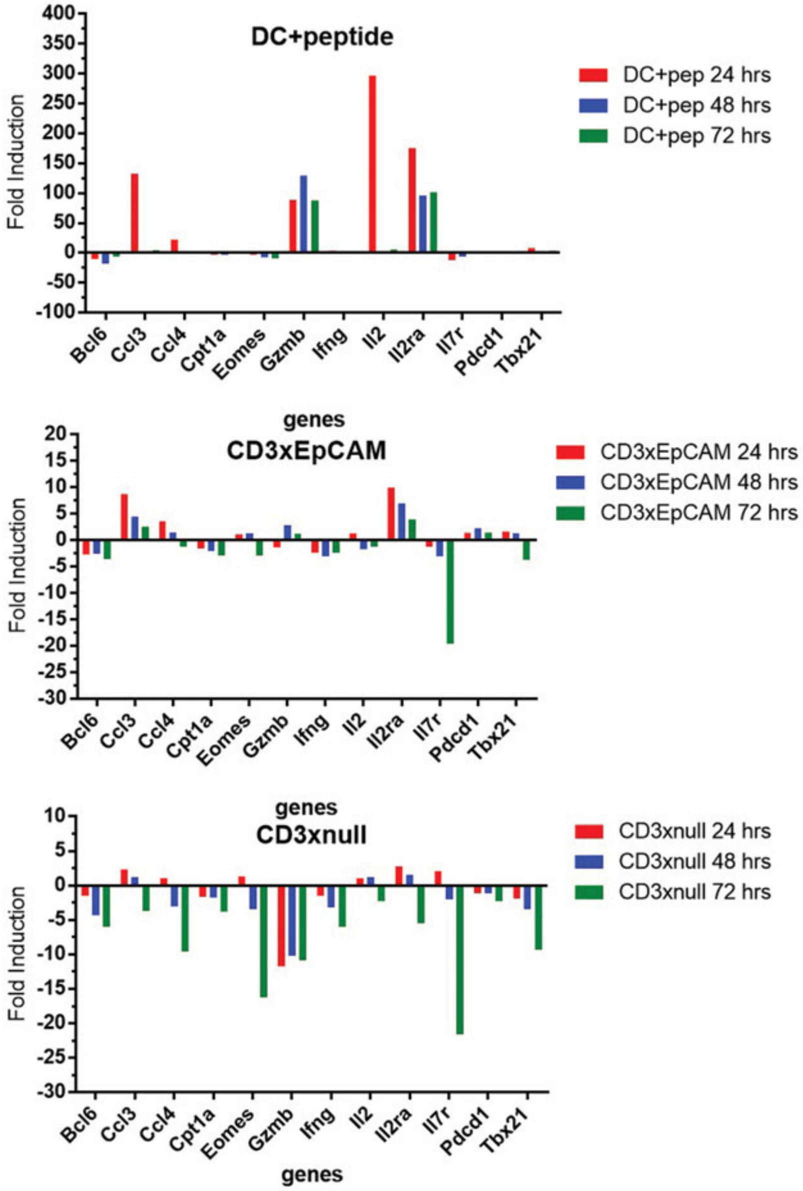

SUPPLEMENTARY FIG. S2. OT1 T cells were activated and cytokine secretion was measured as described in Figure 3. Cytokine secretion of OT1 activated by DC loaded with SIINFEKL peptide is plotted in red. EpCAM, epithelial cell adhesion molecule; DC, dendritic cell.
